# Synovial Tissue Sampling in Rheumatological Practice—Past Developments and Future Perspectives

**DOI:** 10.3389/fmed.2019.00004

**Published:** 2019-01-29

**Authors:** Frances C. Humby

**Affiliations:** William Harvey Research Institute, Barts and The London School of Medicine and Dentistry, Queen Mary University of London, London, United Kingdom

**Keywords:** synovium, biopsy, ultrasound, arthroscopy, rheumatoid arthritis

## Abstract

Synovial biopsies are performed in routine clinical care in order to refine diagnosis as well as within a research setting. Progress in the development of minimally invasive synovial sampling methods in the last century has accelerated and facilitated novel insights into disease pathogenesis. This review discusses the development of synovial biopsy techniques as well as examining the three currently most commonly used approaches: arthroscopic, blind needle biopsy and ultrasound guided approaches. It also highlights major research advances driven through synovial research and considers future developments.

## Introduction: Historical Perspective

**Figure 1:**
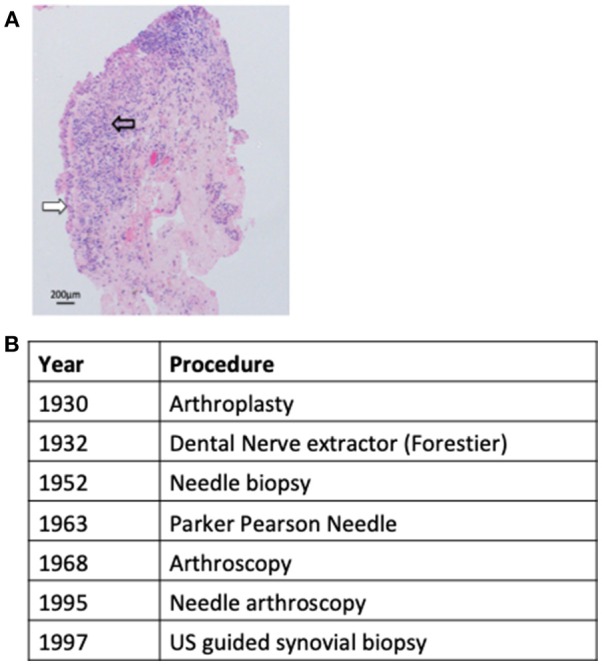
Synovial sampling techniques. **(A)** Representative image of RA synovial tissue demonstrating hypertrophy of lining layer (white arrow) and sublining infiltration by lymphocytes (open arrow). **(B)** The development of minimally invasive synovial sampling techniques.

Synovial tissue lines the diarthrodial joints, tendon sheathes, and bursae, and functions to supply nutrients to the avascular cartilage and to lubricate the joint. In the clinical setting synovial tissue sampling is infrequently required to exclude either infection, when insufficient information is gained from sampling of synovial fluid or peripheral blood, or to refine the diagnosis of an inflammatory synovitis through identifying conditions such as sarcoid (1), Behcets (2), or pigmented villonodular synovitis (3). However, since the term rheumatoid arthritis (RA) was first proposed by Garrod (4) synovial tissue analysis has been utilized as a research tool to examine disease pathogenesis and/or dissect pathogeneic processes determining prognosis and/or response to therapeutic intervention. However, early research efforts in this regard were hampered by access to synovial samples derived from only post mortem specimens or open arthrotomy and thus end stage disease. Although the development of arthroplastic surgery in the early 1930s began to provide a more consistent source of synovial tissue concerns that these samples might not be truly representative of RA pathogenesis were confirmed by later reports that demonstrated significant differences in synovial cellular infiltrate between established and end stage disease (5). Notwithstanding these limitations an observed diversity in synovial histopathological characteristics between patients was noted early on and fuelled efforts to develop novel less invasive methods to sample synovial tissue and examine whether such diversity translated to significance differences in clinical phenotypes. One of the first attempts to develop a minimally invasive sampling technique was by Forrestier who described the application of a modified dental nerve extractor inserted into joints through a larger needle to sample synovial tissue (6). However, formal reports of the method were never published and therefore the technique not translated to clinical practice. Subsequently an approach applying the insertion of a percutaneous needle inserted via a trochar to perform punch biopsies of synovial tissue was reported with success rates approaching 86% for sampling synovial tissue (7–9). However, due to the requirement for an incision and the insertion of a relatively large instrument, although significant complications were not reported, considerable soft tissue trauma was inevitable and this approach therefore not widely adopted. Despite this by 1960 joint features such as histological synovitis, proliferating invasive pannus and cartilage erosions were well described (10). The next major advance arrived with the development of the Parker-Pearson needle in 1963 which utilized a small bore 14G needle and did not require a skin incision (11). A case series of 125 patients documented a success rate of >95% in sampling synovial tissue and moreover demonstrated its safety in this context (11). The subsequent application of the Parker-Pearson needle biopsy or a modification of it (12, 13) led to significant progress in the understanding of RA pathogenesis with reports describing synovial lining layer infiltrates (14) as well as histopathological features of early synovitis (15) (Figure 1A) and remained the instrument of choice for acquiring synovial tissue for diagnostic or research purposes until the 1980s. However, blind needle biopsy was primarily used for sampling synovial tissue from knee joints and was not a useful technique for joints with limited synovitis (16). Thus the transfer of arthroscopy from a primarily diagnostic tool used by orthopedic surgeons to rheumatology research in the 1980s particularly with utilization of smaller bore needle arthroscopes, which permitted access to joints other than the knee and those with minimal synovitis, offered significant advantages, and was readily adopted by the academic rheumatology community (17). Despite this a number of issues associated with arthroscopy such as requirement for highly specialist training, dedicated space and equipment and relatively high cost limited the adoption of arthroscopy outside of large academic rheumatology centers. However, the development of musculoskeletal ultrasound (US) as a diagnostic and management tool for patients with inflammatory arthritis in the mid 1990s presented the opportunity to overcome these limitations by guiding minimally invasive biopsy instruments to synovial tissue via live ultrasound images. Two US-guided biopsy techniques have been reported firstly applying a semi-automatic needle (18) and latterly using a portal and forceps approach (19) to sample synovial tissue. Efforts to validate US-guided biopsy have demonstrated that it appears to be well tolerated (20), able to access a wide range of synovial joints (20–22) provide good quality and quantity of synovial tissue (23) and when applying a semi-automatic needle able to access joints with minimal synovitis (21). Such data therefore supports the current uptake of the technique into both clinical trial protocols as well as routine clinical care (24). The development of synovial sampling techniques over the last century is summarized in Figure 1B.

## Overview of Biopsy Techniques

At present there are broadly three techniques used to sample synovial tissue, which will be discussed briefly below.

### Blind Needle Synovial Biopsy

This is performed following administration of local anesthesia to the skin and subcutaneous tissues up to the joint capsule. Following standard aseptic techniques a trochar is inserted into the joint capsule through which a 14G Parker-Pearson needle is positioned to retrieve synovial tissue. Although most frequently performed on the knee joint, biopsy of the shoulder, wrist, ankle, and elbow has been described and with the introduction of a modified short 2.5 cm needle synovial tissue within the metacarpal phalangeal joints (MCP) has been sampled (25). Sampling of synovial tissue from joints with minimal or no inflammation has also been reported with installation of isotonic saline solution into the joint space prior to biopsy (26, 27) although success rates for successful sampling are lower (16). A comparative study of synovial tissue obtained from clinically active joints using either blind needle biopsy or under direct vision with arthroscopy demonstrated good correlation in terms of microscopic measures of inflammation (27). However given the technical difficulties in successful sampling of synovial tissue from joints with little or no synovitis current recommendations suggest its application should be restricted to diagnostic procedures or cross sectional studies of patients with active arthritis (28). The benefits of blind needle biopsy are that it is technically simple, does not require specialist equipment and is safe (Table 1).

**Table 1:** Considerations for selection of biopsy technique.

	**Arthroscopic**	**U5-NB**	**US·P&F**	**Blind needle biopsy**
Synovial sampling success rates	+++	+++	+++	+++
Technically simple	+	++	++	+++
Patient acceptability	++	++	++	++
Suitable for serial biopsies	+++	+++	+	+
Cost	+++	++	++	+
Suitable for large or small joints	++	+++	+++	+

### US Guided Synovial Biopsy

US-guided synovial biopsy can be performed using either a portal and forceps approach or using a semi-automated needle. Both approaches use standard aseptic protocols and require the installation of local anesthesia to the soft tissues up to the joint capsule and into the joint space. If applying a portal and forceps approach a percutaneous sheath introducer is inserted into the joint under US guidance and either a rigid or flexible forceps introduced to sample synovium (18). Similarly when using a semi-automated needle the closed needle is inserted into the joint and directed to an area of synovium under US guidance (19, 20). The throw of the needle is then opened and synovial tissue sampled. The needle is repeatedly introduced into the joint for multiple biopsy pieces. Although the most recently developed of the available sampling techniques there is an increasing data set to demonstrate its safety, tolerability, and success in reliably sampling synovial tissue both in large and small joints (20, 21, 23) (Table 1). In addition serial sampling of joints is feasible although the quantity of tissue for histological and/or molecular analysis decreases dependent on the degree of pre-biopsy US synovitis (20, 21).

### Arthroscopic Synovial Biopsy

Under the supervision of rheumatologists arthroscopic synovial biopsy is in general performed using a small bore (1–2.7 mm) arthroscope under general or regional anesthesia as a day case procedure. It is technically the most complex of the synovial sampling procedures available and requires two portals. Arthroscopy also requires a dedicated procedure room or theater space and two operators. It does however have a number of advantages including capacity to be performed in MCP, wrist, ankle and knee joints as well as in joints with minimal or no synovitis with excellent success rates for obtaining synovial tissue (29–31). There is also extensive data evaluating its safety including a study evaluating 15,682 procedures performed by rheumatologists (17) demonstrating equivalence in complication rates to those performed by orthopedic surgeons. Furthermore it has been demonstrated to be well tolerated by patients (32). Thus despite the increased training requirements and cost associated with arthroscopic sampling it remains the gold standard procedure for synovial sampling within clinical trials (28).

## Selection of Appropriate Synovial Sampling Technique

Historically data examining performance of synovial biopsy techniques was frequently performed in isolation with little opportunity to compare techniques (20, 22, 32) and thus guide selection of ideal method for a specific setting. Such comparative analyses became increasingly important with the advent of US-guided synovial biopsy, which although readily adopted by the rheumatology community at least initially was not validated against the gold standard arthroscopic approach. In order to tackle these issues validation measures for US-guided biopsy were defined at OMERACT 2014 (33) and since then have steadily begun to be addressed. For example a retrospective analysis of evaluation of 159 biopsy procedures suggested that US-guided procedures, though not those performed using blind needle biopsy, were as successful as arthroscopic in retaining sufficient synovial tissue for histological and molecular analyses (23). In addition recent data examining safety and tolerability of synovial biopsy in a cohort of 524 patients under arthroscopic or US-guided biopsy procedures suggested no differences in outcomes (34). Importantly two large scale biopsy based multicentre international clincial trials, the National Institute Health Research funded Response, relapse, resistance to rituximab (R4RA) (Trial)^1^ and the Arthritis Research-UK (vs. Arthritis)/Medical Research Council funded STratification of Biologic Therapies for RA by Pathobiology (http://www.matura-mrc.whri.qmul.ac.uk)^2^ are due to report outcomes including performance of biopsy techniques in 2019/2020 and will provide the first prospective data sets from randomized controlled clinical trials in which to evaluate performance of both arthroscopic and US-guided biopsy techniques. Considerations for selection of appropriate biopsy technique is summarized in Table 1.

## Major Research Outcomes

Since synovial tissue was identified as the target tissue in RA it's analysis has led to invaluable insights into disease pathogenesis, in addition to the identification of potential therapeutic targets. Furthermore, with the advent of an era of personalized medicine understanding mechanisms of drug response/resistance as well as defining disease prognosis have been major areas of research focus. There are many examples that have been reviewed extensively elsewhere (35). For example the identification that lymphocytic aggregates capable of functioning as ectopic germinal centers and producing disease specific antibodies within synovial tissue in approximately 30% of patients with RA has identified mechanisms driving local autoimmunity (36) and furthermore such structures have been identified as putative biomarkers of response to TNF inhibition (37). Work evaluating synovial tissue response to therapeutic intervention also identified synovial sublining macrophages as key mediators of RA pathogenesis through demonstrating consistent statistically significant reduction in infiltration following therapeutic response (38) an effect that was consistent between centers and across therapies (39). Sublining macrophage number has been translated to a research tool to identify clinical efficacy of novel drugs in early stage clinical development and validated as an outcome marker by OMERACT (39). Potential biomarkers in early arthritis include differential infiltration by CD22+ve B cells and CD38+ plasma cells in patients with early arthritis differentiating RA vs. non RA inflammatory arthritis (40). More recent developments include the identification of joint specific methylation and transcriptomic signatures of synovial fibroblasts (41, 42) providing a potential mechanism to explain both RA joint distribution and differential joint specific therapeutic responses.

## Future Developments

Reliable access to synovial tissue from patients with inflammatory arthritidies is becoming increasingly feasible largely due to the advent of minimally invasive US-guided procedures. Such approaches should facilitate the rapid translation of synovial biomarkers to routine clinical practice once identified. However, further validation of US guided procedures are required including the evaluation of procedures along with arthroscopic within the context of prospective large scale randomized controlled trials with robust reporting measures for defining successful sampling as well as capturing adverse events and patient tolerability in standardized patient cohorts. In addition training requirements for rheumatologists undertaking such procedures, such as have been developed for arthroscopic synovial sampling, need developing.

## Author Contributions

The author confirms being the sole contributor of this work and has approved it for publication.

### Conflict of Interest Statement

The author declares that the research was conducted in the absence of any commercial or financial relationships that could be construed as a potential conflict of interest.

## Footnotes

^1^Trial, R. Available online at: http://www.r4ra-nihr.whri.qmul.ac.uk

^2^Available online at: http://www.matura-mrc.whri.qmul.ac.uk STRAP trial.
